# Capsule Closure of Periportal Capsulotomy for Hip Arthroscopy

**DOI:** 10.1016/j.eats.2022.02.018

**Published:** 2022-06-21

**Authors:** Rami George Alrabaa, Abhishek Kannan, Alan L. Zhang

**Affiliations:** Department of Orthopaedic Surgery, University of California – San Francisco, San Francisco, California, U.S.A.

## Abstract

Multiple approaches for management of the hip capsule during hip arthroscopy for femoroacetabular impingement syndrome have been reported. Capsular closure is advocated in the setting of larger capsulotomies, including interportal and T-capsulotomies, to reduce the risk of iatrogenic instability or microinstability of the hip. The periportal capsulotomy technique has been described for conservative management of the capsule that would not necessitate closure. However, hip arthroscopy for patients with ligamentous laxity or joint hypermobility may warrant capsule closure or plication even with use of conservative capsulotomy techniques. We introduce a technique for closure of periportal capsulotomy as a means to repair or plicate the hip capsule in the at-risk hypermobile patient.

Hip arthroscopy has become the standard in the surgical management of femoroacetabular impingement syndrome (FAIS) and serves as a minimally invasive, less morbid, effective means of addressing bony deformity and chondrolabral pathology. Multiple approaches for management of the hip capsule during arthroscopy have been reported, including interportal capsulotomy,^1^ T-capsulotomy,^2^ and periportal^3^^,^^4^ capsulotomy. Capsular closure is advocated in the setting of larger capsulotomies such as interportal and T-capsulotomies, to reduce the risk of iatrogenic instability or microinstability.^5^^,^^6^ For more conservative capsule management approaches such as periportal capsulotomy, capsule closure is not needed in the general FAIS population without joint hypermobility or dysplasia.^3^ While there remains no consensus regarding capsular management during hip arthroscopy, there remains a subset of the patient population who may benefit from meticulous closure.

Generalized ligamentous laxity (GLL) is defined as supraphysiologic range of movement and is measured by the Beighton score.^7^ While the hip joint’s osseous anatomy imparts inherent stability, capsular management becomes a crucial consideration in patients with a Beighton score of ≥4. In a retrospective review of prospectively collected data, Saadat et al.^8^ demonstrated patients with GLL undergoing hip arthroscopy for FAI are generally younger, have lower body mass index, and are more often female. The study also found patients with greater preoperative Beighton scores had greater hip range of motion and smaller intraoperative labral size and tear dimensions. Maldonado et al.^9^ reported improved patient-reported outcomes and visual analog scale scores at 2-year postoperative for patients with GLL treated with capsular plication, with outcomes comparable with the non-GLL cohort. The association between GLL and microinstability has been proposed,^8^ and capsular plication and closure may be becoming the standard in hip arthroscopy.^6^^,^^10^, ^11^, ^12^ In an at-risk population, capsular closure is crucial to restoration of hip stability and improved clinical outcomes.

Although it has been reported that conservative capsule management with periportal capsulotomies do not necessitate closure in the general FAIS population, capsule repair or plication may be considered in certain at-risk individuals with ligamentous laxity or joint hypermobility. In this Technical Note, we describe our technique for capsular closure of periportal capsulotomy.

## Surgical Technique (With Video Illustration)

The patient is positioned supine on a traction table for hip arthroscopy. Bilateral feet and legs are well padded and positioned into the boots that allow for traction and dynamic limb positioning. A perineal post or “post-less” leg traction systems also can be used. After the operative limb is prepped and draped, it is placed in neutral rotation and an air arthrogram is performed to decompress the negative pressure and suction seal of the joint to allow for adequate traction. After adequate traction is applied, the anterolateral portal (ALP), which is the main viewing portal, is first established under fluoroscopic guidance. A 70° arthroscope is introduced through the ALP and a mid-anterior portal (MAP) is established under direct arthroscopic visualization along with fluoroscopic guidance.

**Fig 1:**
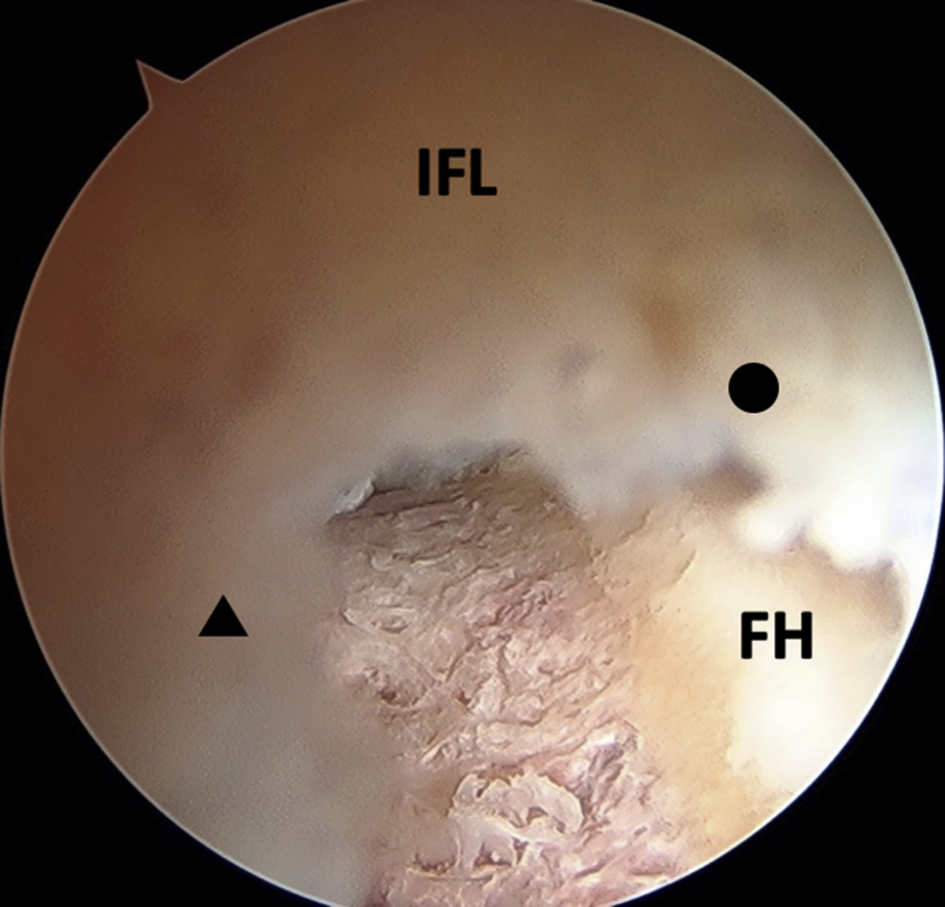
Arthroscopic view of a left hip from the ALP showing the integrity of the iliofemoral ligament remains intact with creation of periportal capsulotomies. The arthroscope is withdrawn so the proximal leaflet (black circle) and distal leaflet (black triangle) of the ALP capsulotomy are shown. (ALP, anterolateral portal; FH, femoral head; IFL, iliofemoral ligament.)

Periportal capsulotomies of the ALP and MAP are performed as previously described.^4^ The ALP is placed in the transition zone or soft spot between with iliofemoral and ischiofemoral ligaments, which we term the “capsular interval of the hip.” The MAP is placed in the center of the iliofemoral ligament. A radiofrequency ablation device (ArthroCare; Smith & Nephew, Andover, MA) is used to dilate both the ALP and MAP portals in line with each other or in the same plane as an interportal capsulotomy would be performed. The ALP is dilated to 6 to 7 mm whereas the MAP is dilated up to a width of 10 mm, which allows for unrestricted introduction and movement of cannulas and arthroscopic instruments intraarticularly (Fig 1). The MAP capsulotomy is larger as it is the main instrumentation portal. An 8- × 90-mm disposable plastic cannula (Smith & Nephew) is placed through the MAP for instrumentation while the ALP is the main viewing portal.

Arthroscopic management of intraarticular hip pathology is then carried out based on the pathology. Pincer lesions are resected with a 5.5-mm round burr (Stryker, Kalamazoo, MI) mainly through the MAP. Labral repair in our practice is performed mostly through the MAP using all-suture anchors with flexible drills for placement (Pivot NanoTack Flex; Stryker). Once pincer and labral pathology is address, traction is released and femoroplasty of the CAM lesion is performed in a systematic fashion as previously described.^13^

**Fig 2:**
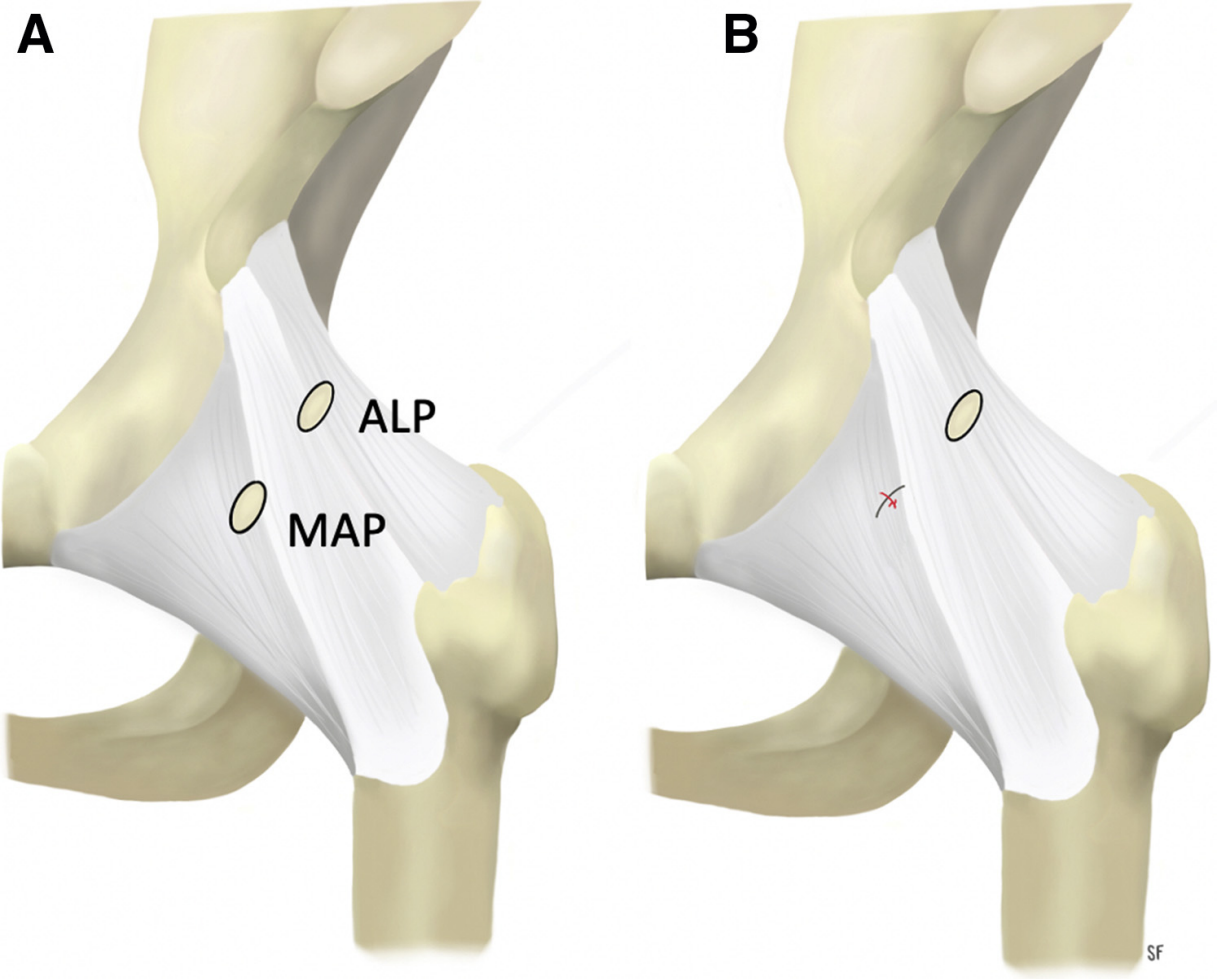
Illustration of the anterior hip capsule of a left hip depicting the locations of the anterolateral and mid-anterior portals (A). The anterolateral portal lies at the capsular interval in the transition zone between the iliofemoral and ischiofemoral ligaments and does not necessitate closure. (B) Illustration after closure of the MAP. (ALP, anterolateral portal; MAP, mid-anterior portal.)

**Fig 3:**
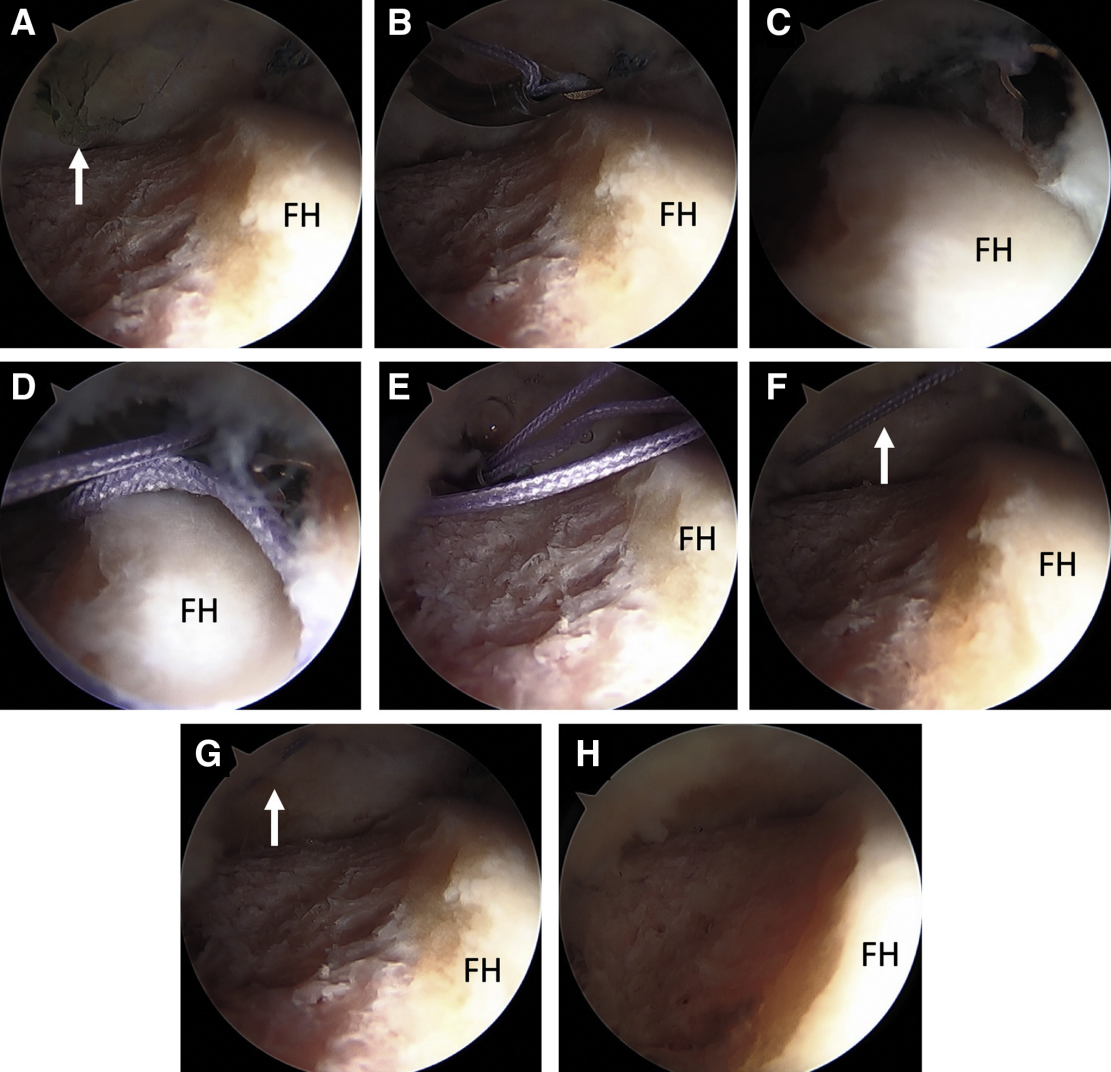
Arthroscopic view of a left hip in the supine position through the ALP demonstrating capsular closure of a periportal capsulotomy of the MAP. (A) A plastic disposable working cannula that was used for the labral repair and femoroplasty is shown within the joint (white arrow). This cannula is withdrawn superficial to the capsular tissue before performing capsular repair to allow for outside-in passing of sutures. (B) A 70° suture passer (SlingShot; Stryker) is shown within the joint introduced through the MAP loaded with #2 nonabsorbable suture (ORTHOCORD; DePuy Synthes). Note that the cannula has been withdrawn superficially and is no longer visualized intra-articularly. (C) The suture passer penetrates the proximal capsular leaflet from outside-in. (D) The #2 suture is deposited into the joint. (E) The suture passer then penetrates the distal leaflet of the capsule and retrieves the suture from within that joint that was already passed through the proximal capsular leaflet. (F) Both limbs of the suture are now emerging through the MAP and arthroscopic view from within the joint shows the simple suture configuration (white arrow). The suture limbs are tied together from the outside through the MAP using an arthroscopic knot pusher. (G) Note the tensioning of the suture (white arrow) and closure of the peripheral space with knot tying. (H) View through the ALP after completed capsular closure of a periportal capsulotomy of the MAP. Note the decrease of space in the peripheral compartment of the hip. (ALP, anterolateral portal; FH, femoral head; MAP, mid-anterior portal.).

Although capsular closure is not necessary after periportal capsulotomy in most patients, it can be considered in hypermobile patients with GLL to close or plicate the MAP, as this portal is centered in the iliofemoral ligament. The ALP, In contrast, does not require closure as it rests in the capsular interval of the hip which is an anatomically thin transition zone and it undergoes minimal dilation with periportal capsulotomy (Fig. 2A). After completion of arthroscopic FAI treatment, the leg is placed in neutral rotation and flexion and capsule closure of the periportal capsulotomy can be performed (Video 1). The 70° arthroscope in the ALP is used to view the closure intra-articularly underneath the hip capsule. The 8-mm plastic working cannula that was used for labral repair and femoroplasty in the MAP is withdrawn superficial to the hip capsule to the level of the musculature (Fig 3A). This allows the 70° SlingShot (Stryker) suture passer to have increased maneuverability and enables outside-in passing of the suture (Fig 3B). A high tensile strength nonabsorbable suture is loaded onto the SlingShot suture passer for capsular closure. In our practice, #2 ultra-high molecular weight polyethylene suture is used (ORTHOCORD; DePuy Synthes, Warsaw, IN). The slingshot is used to first penetrate the proximal capsular leaflet from outside-in through the proximal/medial aspect of the MAP (Fig 3C), and the #2 suture is deposited into the joint (Fig 3D). Intra-articular visualization of this steps ensures there is no damage to the labrum/labral repair from the slingshot. The suture passer is then withdrawn from the proximal leaflet; care is taken not to withdraw the suture that was passed along with the passer. Next, the suture passer is used to penetrate the distal capsular leaflet from outside-in, and the suture that was passed through the proximal leaflet is retrieved (Fig 3E). This creates a simple suture configuration through the periportal capsulotomy. If plication of the capsule is desired, then a more distal entry point can be made with the slingshot so a bigger “bite” is taken of the distal aspect of the capsulotomy to advance the tissue. The suture limbs are then tied from outside through the disposable working cannula using an arthroscopic knot-pusher and alternating half-hitches. The arthroscope is left deep to the capsule to visualize the closure as the knots are tied and the peripheral compartment space is tightened (Fig 3 F-H). Figure 2B shows an illustration of the closed MAP portal.

Pearls and pitfalls (Table 1) as well as advantages and disadvantages (Table 2) of this technique are summarized.

**Table 1:** Pearls and Pitfalls of Periportal Capsulotomy Closure for Hip Arthroscopy

Pearls	Pitfalls
• Positioning of the lower extremity in neutral hip flexion provides natural resting tension of the capsule and prevents overtightening of the capsule. • Withdrawal of the working cannula in the MAP superficial to the capsular layer allows for adequate maneuverability of the suture passer device for capsular closure. • Begin with passing suture through the proximal capsular leaflet followed by the distal leaflet. • Use of a curved suture passer (we prefer the 70° SlingShot suture passer) allows for easier and more controlled penetration and passage of suture through the capsular tissue. • Visualize the capsule intraarticularly during capsular closure to ensure no damage to the labrum from suture passing and adequate tension is restored.	• Positioning of the lower extremity in excessive hip flexion may decrease visualization and cause overtightening of the hip capsule. • Failure of withdrawal of the working cannula superficial to the full thickness of the capsule hinders maneuverability and the ability of the suture passer to achieve full-thickness penetration of the capsular leaflets. • Failure to arthroscopically visualize the capsule intraarticularly during capsular closure may result in unrecognized labral injury from suture passing, inadequate tensioning of the capsular closure or unrecognized propagation of the capsulotomy into a full interportal capsulotomy.

MAP, mid-anterior portal.

**Table 2:** Advantages and Disadvantages of Periportal Capsulotomy Closure for Hip Arthroscopy

Advantages	Disadvantages
• Only one simple configuration suture is needed for periportal capsular closure. • Decreased risk for postoperative instability or microinstability with capsular closure in hypermobile patients.	• Increased cost to case with additional instruments needed compared with no capsular closure. • Capsular closure of a periportal capsulotomy in patients without hypermobility may lead to potential excessive constraint.

## Discussion

Described capsular entry techniques in hip arthroscopy include periportal, interportal, and T-capsulotomies, all of which have the potential to provide varying levels of visualization and access to central and peripheral compartments. The current literature lacks a clear consensus regarding risks and benefits of various capsular management strategies and their respective biomechanical and clinical outcomes. Some authors have shown capsular deficiency may be associated with revision surgery and iatrogenic instability.^5^^,^^6^^,^^14^^,^^15^ While some studies demonstrate repair provides improved hip stability,^16^ biomechanics,^16^^,^^17^ and range of motion,^18^ others suggest no adverse clinical consequences when the capsule is left unrepaired.^11^^,^^19^^,^^20^

Cadaveric studies demonstrate hypermobility after large interportal (4-6 cm) or T-capsulotomies.^18^^,^^21^ The iliofemoral ligament (Y ligament of Bigelow), the strongest of 3 capsular ligaments, is transected during interportal or T-capsulotomy, eliminating static restraint to hip extension and anterior translation.^22^ As the pseudonym implies, the Y-shape of the iliofemoral ligament describes a proximal convergence of fibers resulting in a thin transition zone, or capsular interval, between it and the ischiofemoral ligament where the ALP is made (Fig 3). The ALP, used primarily for viewing, is dilated only 6 to 7 mm as described for periportal capsulotomy.^4^ It is located in this capsular interval and consequently does not necessitate closure. Closure of the working MAP within the substance of the iliofemoral ligament provides leaflet apposition for sound healing and restoration of intrinsic static restraint.

Instability following hip arthroscopy is multifactorial with limited reports in the literature. In a systematic review of subluxation and dislocation following hip arthroscopy, Duplantier et al.^15^ identified 10 articles with 11 patients, 9 of whom suffered dislocation and 2 subluxations. Of the 8 reported interportal capsulotomies, only 2 were repaired. Wuerz et al.^18^ demonstrated capsulotomies were accompanied by increased joint mobility and showed restored range of motion when compared with the intact condition. With respect to short-term clinical outcomes, Frank et al.^23^ found improved outcomes and patient satisfaction following complete repair in comparison to partial repair at 2 years’ postoperative. McCormick et al.^24^ reviewed patients undergoing revision hip arthroscopy and after excluding patients with residual FAI as cause for revision, the investigators found 9 of 25 patients with hip capsule abnormalities, 7 of whom had capsular defects detected on MRA. Notably, no patients with capsular closure at the index procedure were found to have residual capsular defects. In another study, of 229 patients undergoing revision hip arthroscopy, Selley et al.^25^ found revision for instability as the second most common indication at 14.8%. Of these, 34.2% were attributed to atraumatic capsular deficiency. While residual FAI remains the most common indication for revision arthroscopy, capsular complications are increasingly recognized as an etiology for failed index procedures. With increasing hip arthroscopy use in the United States, capsular management will become ever more crucial to clinical efficacy and patient outcomes.

Periportal capsulotomy, as previously described,^4^ has been shown to not necessitate capsule closure in patients with FAI without ligamentous laxity.^3^ However, in patients with hypermobility, even partial injury to the iliofemoral ligament may result in microinstability. It is in this at-risk patient population where periportal capsulotomy closure may be considered, with particular attention to closure of the mid-anterior capsulotomy.

In conclusion, periportal capsulotomy for hip arthroscopy in patients without ligamentous laxity does not necessitate capsule closure but for patients with joint hypermobility a simple technique to repair or plicate the periportal capsulotomy can be used.

## Supplementary Data

ICMJE author disclosure forms

Video 1 This video shows capsule closure of the periportal capsulotomy of the mid-anterior portal (MAP) in a left hip.
